# A Case of Pyocolpos in a Postmenopausal Woman With a History of Lichen Sclerosus and Postmenopausal Bleeding

**DOI:** 10.7759/cureus.61507

**Published:** 2024-06-01

**Authors:** Amila J Rubasinghe, Bramara Guruwadayarhalli, Aderemi Alalade, Kyin Yu Maw

**Affiliations:** 1 Obstetrics and Gynaecology, Wrexham Maelor Hospital, Wrexham, GBR; 2 Obstetrics and Gynaecology, The Grange University Hospital, Cwmbran, GBR

**Keywords:** estrogen replacement therapy, post menopause, lichen sclerosus, post-menopausal bleed, pyocolpos

## Abstract

Pyocolpos refers to the buildup of pus within the vaginal cavity. Pyocolpos in the background of lichen sclerosis and postmenopausal bleeding (PMB) has not been previously described. A 69-year-old para 3 patient presented with a history of PMB with a long-standing history of lichen sclerosis. The vaginal examination was impossible due to vaginal adhesions. Vulval appearances revealed the loss of the clitoral architecture. Further imaging revealed an endometrial thickness of 4-5 mm, a focal abnormality within the posterior ectocervix compatible with a hemorrhagic cystic lesion distending the posterior fornix, and some free fluid within the pelvis. A hysteroscopy was abandoned as the vagina was completely obliterated. After a multidisciplinary assessment, the patient had a total abdominal hysterectomy, and the presence of a pyocolpos was noticed at the opening into the vault. We could not find any previous case reports of pyocolpos that are associated with lichen sclerosus. The long-standing history of lichen sclerosus may have caused an obstruction of the outflow tract, which was secondarily infected and slowly progressed into the formation of pyocolpos. Other management options could have been explored if the diagnosis of pyocolpos had been made preoperatively. Pyocolpos should be considered in patients with a history of a long-standing lichen sclerosus who present with abdominal pain and a pelvic mass on imaging.

## Introduction

Pyocolpos is defined as the accumulation of purulent material in the vaginal cavity [1], which may be secondary to multiple etiologies. It is a rare gynecological diagnosis. The number of reported cases is limited and is mostly secondary to infections among neonates and children with congenital anomalies of the genitourinary tract. Pyocolpos in the background of lichen sclerosis and postmenopausal bleeding (PMB) has not been previously described.

## Case presentation

The patient was a 69-year-old para 3 who presented to the outpatient clinic with a history of PMB. She had a background of lichen sclerosus and had undergone separation of labial adhesions and Fenton’s repair at the age of 65. She managed her lichen sclerosus with topical ultrapotent steroids (Dermovate) and emollients, and at the time of presentation, she was on a maintenance dose of daily emollients and twice-weekly topical steroids. She had also undergone a course of topical estrogen treatment two years prior. She managed flare-ups with short courses of daily topical steroids. Regarding her past medical history, she was receiving treatment for diabetes and hypertension. Her diabetes was not well controlled, and she had developed microvascular complications such as diabetic retinopathy.

She had presented to the emergency unit two weeks earlier with severe vaginal and abdominal pain. However, there was no history of vaginal discharge, urinary symptoms, or fever. Blood tests, including a full blood count and CRP, were normal (Table 1). Urine microbiology revealed* Escherichia coli*, and a vaginal swab was positive for group B Strep (GBS), which is considered normal vaginal flora. No diagnostic imaging was offered during this admission. She was managed symptomatically and treated with nitrofurantoin for this episode.

**Table 1:** Investigation results of the initial hospital admission

Investigation	Results	Reference range
White blood cell count	4.6 × 10^9^/L	(4.0-11.0) × 10^9^/L
Neutrophil count	2.4 ×10^9^/L	(1.7-7.5) × 10^9^/L
Lymphocyte count	1.8 × 10^9^/L	(1.0-4.5) × 10^9^/L
CRP	7 mg/L	<5 mg/L
Serum creatinine	56 umol/L	(46-92) umol/L

**Figure 1:**
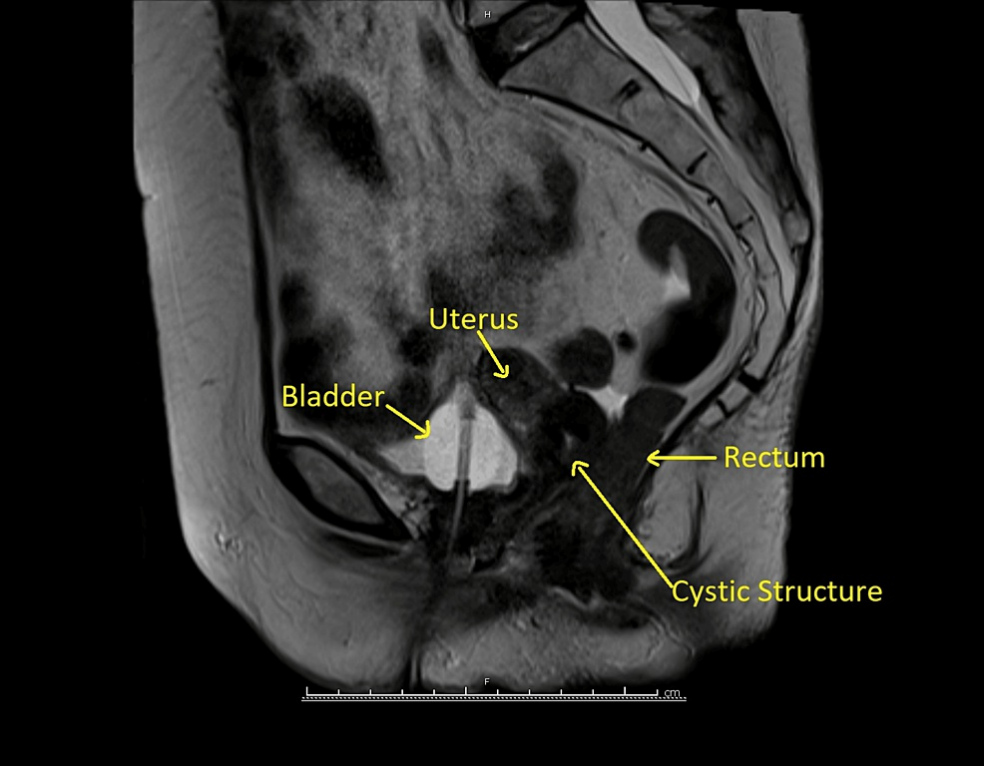
MRI pelvis: midsagittal view

Pelvic examination and vulval appearance revealed a loss of the clitoral architecture consistent with lichen sclerosus. However, a vaginal examination was impossible due to vaginal adhesions. For the same reason, a pelvic ultrasound was not completed. An MRI of the pelvis showed an endometrial thickness of 4-5 mm and a focal abnormality within the posterior ectocervix consistent with a hemorrhagic cystic lesion distending the posterior fornix. The vagina did not show significant abnormalities, and the lower rectum/anorectal junction exhibited some mild, non-specific thickening. There was some free fluid within the pelvis, but no enlarged lymph nodes (Figure 1).

The patient consented to an examination under anesthesia (EUA) and hysteroscopy. Findings during the EUA included an atrophic vulva and vagina and fused labia minora consistent with lichen sclerosus. The vagina was completely obliterated, and the cervix was not visualized; therefore, the hysteroscopy was abandoned. She was referred for discussion at the gynecological oncology multidisciplinary team review. Following this, treatment in the form of a hysterectomy, a bilateral salpingo-oophorectomy, and an omental biopsy was offered.

During surgery, findings revealed a normal, atrophic uterus, ovaries, fallopian tubes, and normal intra-abdominal organs. At hysterectomy, the infundibular and uterine pedicles were successfully ligated. During colpotomy, a large collection of brownish, non-smelly purulent material was observed. A sample of the material was obtained for microbiology. The large bowel was inspected, and a bowel survey was conducted, ruling out bowel injury. Subsequently, the hysterectomy was completed, and the vaginal vault was closed. The abdominopelvic cavity was thoroughly irrigated, followed by standard abdominal closure. Postoperatively, she received antibiotics (a single dose of gentamicin, cefuroxime, and metronidazole for a total of seven days) and had an uneventful recovery. The postoperative surgical histology was normal, with inactive and benign endometrium, cervix, and unremarkable adnexal tissue, along with some adenomyotic changes in the myometrium. The pus culture was positive for GBS.

## Discussion

Pyocolpos mainly occurs in situations where secretions get accumulated in the incompletely canalized genital tract (vaginal atresia, vaginal septum, and imperforate hymen), forming a hydrocolpos that secondarily becomes infected [2]; hence, it is mainly seen in the pediatric age group [3]. It has been described in conditions including Laurence-Moon-Bardet-Biedl syndrome [4] and Herlyn-Werner-Wunderlich syndrome [5] (obstructed hemivagina and ipsilateral renal anomaly syndrome). It is rarely seen in adults, including pregnant women [6,7].

Pyocolpos is very rare in postmenopausal women, and the majority of reported cases in this age group occur following colpocleisis [1,8,9]. There was one reported case of spontaneous pyocolpos in an elderly woman [1], and it can be diagnosed with pelvic imaging, including ultrasound scans, CT, and MRI. It is necessary to confirm or rule out a primary pathology such as malignancy during the course of treatment, then drain the pus and prescribe appropriate antibiotics.

In our literature survey, we could not find any previous case reports of pyocolpos that are associated with lichen sclerosus; hence, this will be the first reported case of such an association. The long-standing history of lichen sclerosus may have caused an obstruction of the outflow tract, which was secondarily infected and slowly progressed into the formation of pyocolpos. The significance of local estrogen therapy causing increased cervical mucus secretion into the obstructed space is debatable. PMB, which was associated with pyocolpos, was also not found in the literature. In this case, the cervical lesion that appeared in the MRI was the pyocolpos.

Other management options could have been explored if the diagnosis of pyocolpos was made preoperatively, which might have resorted to a less invasive option, including vaginal drainage under ultrasound guidance, with a close follow-up for increased endometrial thickness or hysteroscopy.

## Conclusions

Pyocolpos should be considered in patients with a history of a long-standing lichen sclerosus who present with abdominal pain and a pelvic mass on imaging. Recognition of this pathological condition should lead to prompt and potentially less invasive intervention with a vulvovaginal approach. However, significant anatomical distortions due to advanced disease changes may make this practically challenging.
